# Efficacy of Bromhexine versus Standard of Care in Reducing Viral Load in Patients with Mild-to-Moderate COVID-19 Disease Attended in Primary Care: A Randomized Open-Label Trial

**DOI:** 10.3390/jcm12010142

**Published:** 2022-12-24

**Authors:** María Luz Vila Méndez, Carmen Antón Sanz, Alicia del Rocío Cárdenas García, Amparo Bravo Malo, Francisco Javier Torres Martínez, José María Martín Moros, María Real Torrijos, José Francisco Javier Vendrell Covisa, Olga Guzmán Sierra, Verónica Molina Barcena, Nuria Viejo Pinero, Carlos Fernández Díaz, Purificación Arroyo Burguillo, Ana María Blanco Gallego, Carmen Guirao Sánchez, Aránzazu Montilla Bernabé, María del Pilar Villanueva Morán, Salvador Juárez Antón, Ángela Fernández Rodríguez, María Ángeles Somoza Calvo, Ernesto Cerrada Cerrada, Gemma Pérez Mañas, Antonio Sánchez Calso, Frida Vallejo Somohano, Carmen Cauqui Díaz, Gloria Viñas Fernández, Jesús Molina París, Marina González Godoy, Gonzalo Lumbreras García, Javier Rosado Martín, Aida Rodríguez Hernández, Sara López Antúñez, Gabriel Vázquez Perfecto, María Concepción Marcello Andrés, Nieves Marina Puente García, Carmen Gil, Ana Martínez, Begoña Soler López

**Affiliations:** 1 Centro de Salud Fronteras, 28850 Torrejón de Ardoz, Spain; 2 Consultorio Alpedrete, 28430 Alpedrete, Spain; 3 Centro de Salud Palma Norte, 28015 Madrid, Spain; 4 Centro de Salud María de Guzmán, 28805 Alcalá de Henares, Spain; 5 Consultorio Brunete, 28690 Brunete, Spain; 6 Centro de Salud Villalba Estación, 28400 Collado Villalba, Spain; 7 Consultorio Collado Mediano, 28450 Collado Mediano, Spain; 8 Centro de Salud Benita de Ávila, 28043 Madrid, Spain; 9 Consultorio de Moralzarzal-EAP Villalba Pueblo, 28411 Moralzarzal, Spain; 10 Centro de Salud Francia, 28943 Fuenlabrada, Spain; 11 Centro de Salud Galapagar, 28260 Galapagar, Spain; 12 Centro de Salud Reina Victoria, 28003 Madrid, Spain; 13 Centro de Salud Dos de Mayo, 28934 Móstoles, Spain; 14 Centro de Investigaciones Biológicas “Margarita Salas” (CSIC), 28040 Madrid, Spain; 15 E-C-BIO, S.L., Las Rozas, 28230 Madrid, Spain

**Keywords:** COVID-19, SARS-CoV-2, bromhexine, standard of care, viral load, primary care

## Abstract

A 28-day randomized open-label multicenter study was conducted to assess the efficacy of bromhexine plus standard of care (SOC) (*n* = 98) vs. SOC alone (*n* = 93) in 191 outpatients with mild-to-moderate COVID-19 in the primary health care setting. Bromhexine three daily doses of 10 mL (48 mg/day) were administered for seven days. The primary efficacy endpoint was the reduction of viral load estimated as the cycle thresholds (Ct) to detect ORF1ab, N Protein, and S Protein genes by RT-qPCR in saliva samples on day 4 as compared with baseline. Ct values of the three genes increased from baseline throughout days 4 to 14 (*p* < 0.001) but significant differences between the study groups were not found. Differences in the percentages of patients with low, medium, and high viral loads at 4, 7, and 14 days were not found either. In summary, treatment with bromhexine plus SCO was associated with a viral load reduction of ORF1ab, N Protein, and S Protein genes at day 4, which was not significantly different than similar viral load reductions observed with SOC alone. The present findings do not seem to favor the use of bromhexine as an antiviral in patients with COVID-19.

## 1. Introduction

Bromhexine is a marketed mucoactive drug currently indicated as a symptomatic treatment of upper respiratory infections. It is an old over-the-counter medication that has been extensively used for decades as a mucolytic agent, it is well-tolerated and safe. The adverse reactions related to the use of bromhexine were of low frequency (≥1/1.000 to <1/100) and include vomiting, diarrhea, nausea, and upper abdominal pain. The spread of COVID-19 has stimulated huge efforts to find active treatments against SARS-CoV-2 infection, either searching for novel molecules or repurposing old drugs [1].

Cell entry of coronaviruses depends on the binding of the viral spike (S) proteins to cellular receptors and on S protein priming by proteases of host cells [2]. It has been shown that SARS-CoV-2 uses the SARS-CoV receptor angiotensin-converting enzyme 2 (ACE2) as the entry receptor and employs cellular transmembrane protease serine 2 (TMPRSS2) for S protein priming [3,4]. Therefore, TMPRSS2 inhibitors approved for clinical use blocking host cell entry might constitute a treatment option for COVID-19. A potential mechanism of action of bromhexine is related to the blockade of virus entry into the cell mediated by the TMPRSS2 receptor [5,6].

There is limited data on the potential role of bromhexine in the management of COVID-19. It is relevant to highlight that bromhexine has been initially identified as a potent inhibitor (IC50 = 0.75 μM) of the transmembrane serine protease 2 (TMPRSS2) of SARS-CoV [5], being involved also in the binding and infection (mainly via a non-endocytotic route) of SARS-CoV-2 to host cells [7]. The probability of success in identifying molecules with antiviral potential is markedly increased by including different phases of the viral replication cycle [8]. Recent studies ruled out that TMPRSS2 inhibition is responsible for the antiviral activity of bromhexine in SARS-CoV-2, as slight antiviral activity is reported in VeroE6 cells, which lack TMPRSS2 in their membranes [4]. Moreover, a multitarget approach of bromhexine to several viral and human proteins may explain its potential efficacy against SARS-CoV-2 [9].

While the assessment of bromhexine clinically in the care of patients with COVID-19 has been encouraged [10,11], only a few clinical studies have been published in the literature. In 111 hospitalized patients with confirmed COVID-19 randomized 1:1 to treatment with bromhexine (8 mg four times daily) or standard treatment lopinavir/ritonavir and interferon beta-1a, there was no difference in clinical improvement within 28 days (primary outcome) as well as in other secondary outcomes including length of intensive care unit (ICU) stay, the average time to hospital discharge, duration of supplemental oxygen, or risk of death by day 28 [12]. In contrast, in another randomized open-label study of 78 patients, the early administration of bromhexine (8 mg four times daily) for 2 weeks in addition to standard therapy reduced the need for ICU admission, intubation/mechanical ventilation, and 28-day mortality [13]. In a randomized open-label study of medical staff actively involved in the care of patients with suspected or confirmed SARS-CoV-2 infection, prophylactic treatment with bromhexine (8 mg three times daily) was associated with fewer participants who developed symptomatic COVID-19 as compared to controls, although differences in positive swab PCR test or signs of clinical infection at day 28 were not found [14]. 

However, the potential efficacy of bromhexine in asymptomatic post-exposure subjects or in patients with mild infection managed in the outpatient setting remains to be determined. As the infective capacity is related to the patient’s viral load, if we were to achieve an antiviral therapy that reduces the viral load and acts on the patient population that has not yet developed symptoms or has developed them recently, we could impact the capability to transmit the virus early, and also delay or prevent the appearance of the first symptoms as well as the disease progression to more severe forms [15]. 

Therefore, the present randomized open-label clinical trial was conducted to assess the efficacy of bromhexine as compared with standard of care (SOC) to reduce the viral load in patients with mild-to-moderate COVID-19 disease attended in the primary healthcare setting. To the best of our knowledge, this is the first comparative drug repositioning study to evaluate the benefits of an old drug in the treatment of infection caused by SARS-CoV-2 in patients with early-stage COVID-19 disease including asymptomatic subjects. 

## 2. Materials and Methods

### 2.1. Study Design and Objectives

This was a phase 3, randomized, open-label, parallel group, controlled, multicenter clinical trial conducted in 19 primary healthcare centers located in the autonomous community of Madrid, Spain. The study period began on 24 February 2022 and finished on 28 July 2022. The duration of the study for each patient was 28 days.

The primary objective was to assess the efficacy of bromhexine plus SOC (active treatment) versus SOC alone (control) in reducing viral load at day 4 from baseline. Secondary objectives included the efficacy of bromhexine plus SOC versus SOC to get negative PCR from baseline. To reduce the intensity and duration of symptoms, to assess the need for medical care, admission to the hospital, and oxygen therapy, the mortality rate through day 28 from baseline, and safety of the active treatment. 

The study was conducted in accordance with the principles of the Declaration of Helsinki and approved by the Medicinal Product Research Ethics Committee of Hospital Universitario Puerta de Hierro Majadahonda (Madrid, Spain) (code 21/2021, approval date 12 December 2021). The study was registered at European Union Drug Regulating Authorities Clinical Trials Database (EudraCT) (number 2021-001227-41). Written informed consent was obtained from all participants.

### 2.2. Participants

Eligible subjects were men or women aged 18 years or older, diagnosed with active SARS-CoV-2 infection confirmed by a positive rapid antigen detection test or a PCR test for viral RNA detection in the presence of compatible symptoms (fever, cough, shortness of breath or difficulty breathing, sore throat, body or muscle pain, fatigue, headache, chills, nasal congestion, loss of taste or smell, nausea or vomiting, and diarrhea). Symptomatic patients were required to have had one or more of the clinical manifestations within the last 72 h, the severity of which was mild or moderate. Exclusion criteria were patients living with a patient who had been enrolled in the present study and continued to be followed over the 28-day study period; patients with severe COVID-19; the presence of diseases that may be affected or interfere with the results of the study (such as active infections other than SARS-CoV-2 requiring systemic therapy, uncontrolled respiratory disorder, prior ischemic heart disease, heart failure or atrial fibrillation, severe renal failure, active or treated malignancy, immunosuppression status, expected elective surgery within 30 days after screening for the study, severe obesity); concomitant treatment with drugs with known antiviral potential; hypersensitivity or intolerance to bromhexine (or any of the excipients); pregnant or breast-feeding women; inability to understand the informed consent; ineligibility as judged by the investigators; and participation in a clinical trial within the last 30 days. All the patients needed to be informed about the study procedures and sign the informed consent form.

### 2.3. Randomization and Intervention

Randomization was generated by an independent technician using a web-based randomization system (http://www.randomization.com, accessed on 22 November 2022). Patients were randomized 1:1 to the active treatment or the control arm according to an allocation sequence in random blocks of 4 and 6 treatments for a total of 10 treatments for each study center. The order of blocks in each group of 10 treatments was also randomized. The allocation concealment was done by electronic database monitoring. After the patient signed the informed consent, the investigator opened the randomization envelopes and assigned the corresponding intervention.

Patients randomized to the active treatment received bromhexine, 3 daily doses of 10 mL (48 mg/day) for 7 days plus SOC therapy. Two bottles of 200 mL (16 mg per 10 mL) were provided to each patient. Since the efficacious daily dose of the active product with viral load reduction capacity was unknown, the maximum labeled dose of the marketed product (16 mg/10 mL 3 times daily equal to 48 mg/day or 30 mL/day) for 7 days was analyzed. No bromhexine dose increase was allowed. Labeling and packing of bromhexine followed the Good Manufacturing Practice (GMP) regulations and local or national regulatory requirements.

The SOC for SARS-CoV-2 infection included acetaminophen 500 mg (1–4 times daily), non-steroidal anti-inflammatory drugs (NSAIDs), symptomatic treatment, and hydration for mild COVID-19. In moderate disease and only in case of suspicion of bacterial coinfection/superinfection, the following should be prescribed: oral azithromycin 500 mg every 24 h for 3 days plus amoxicillin 1 g every 12 h for 7 days, or amoxicillin-clavulanate 875–125 mg every 8 h for 7 days; or alternatively, levofloxacin 500 mg every 12 h on the first day and 500 mg every 24 h for 4 days. Other treatments when required included bronchodilators or inhaled corticosteroids in patients with asthma or chronic obstructive pulmonary disease (COPD), low doses of systemic corticosteroids in patients requiring oxygen therapy, and antithrombotic prophylaxis in patients immobilized or with risk factors for thrombosis [16,17].

### 2.4. Study Procedures

The study included a screening visit (baseline), in which eligibility criteria were confirmed, a complete medical history was taken, a SARS-CoV-2 rapid antigen test was performed, a salivary sample was collected for a SARS-CoV-2 PCR test, a peripheral fasting blood sample was drawn for laboratory analyses, the informed consent was signed, and a diary and the study medication were provided. Patients were instructed on how to take the assigned medications and to complete the diary card, in which the hospitalization criteria were described in plain language.

Telephone contacts were completed on days 1, 4, 7, and 14 after starting treatment. At the end of the study, on day 28, patients were visited at the primary care center. Saliva samples for SARS-CoV-2 PCR assay were collected on baseline and days 4, 7, and 14 at the patients’ homes due to the limitation of medical visits in quarantined patients. In all telephone contacts, pulse oximetry data, heart rate, and temperature recorded by the patient with the study material supplied for that purpose were registered. Questions about the appearance of new symptoms and the severity of symptoms were assessed on a numerical rating (NRS) severity scale of 0 to 10 points (0 = no symptoms, 10 = the most severe symptoms imaginable). Symptoms recorded in the diary card as well as non-prescribed concomitant drugs were communicated to the physician during the telephone calls. Also, the investigator asked the patients if they have experienced any adverse events since the last study contact, and if any exist, recorded them on the “Adverse Event” case report form page and described the event. All adverse events were followed until their resolution or chronicity.

### 2.5. Viral Load

Viral load was determined by the detection of three highly conserved epitope regions within the SARS-CoV-2 pathogenic viral RNA strain, pen reading frame ORF) 1ab (ORF1ab), nucleocapsid N protein (N Protein), and spike S protein (S Protein), in saliva samples on baseline and days 4, 7, and 14 after initiation of treatment. These analyses were performed in a central laboratory (Arquimea Medical, S.L., Leganés, Madrid, Spain). Viral RNA was obtained using the chemagic^™^ Viral DNA/RNA 300 kit H96 from (PerkinElmer España, S.L., Tres Cantos, Madrid, Spain), and purification was carried out using the automated chemagic 360 Instrument (PerkinElmer). RT-qPCR was completed with the TagPath™ COVID-19 CE-IVD RT-PCR Kit (Thermo Fisher, Waltham, MA, USA), and detection of OFR1ab, N Protein, and S Protein was completed in the 7500 Real-Time PCR Instrument (Thermo Fisher) and QuantStudio Real-Time PCR Instrument (Thermo Fisher). The sensitivity and specificity of the platform are >99% and 99.5%, respectively. The viral load was estimated as the number of amplification cycles (cycle thresholds, Ct) to detect genes encoding ORF1ab, N Protein, and S Protein in a single PCR reaction. An RT-qPCR for SARS-CoV-2 was considered positive in the presence of a Ct value lower than 35 for at least two of the three genes analyzed. A higher number of cycles means a lower viral load. Viral load was defined as ‘high’ for Ct values ≤ 25, ‘medium’ for Ct values > 25 and ≤30, and ‘low’ for Ct values ≥ 30.

### 2.6. Definitions

Asymptomatic or pre-symptomatic infection was defined in the presence of a positive diagnostic RT-qPCR test for SARS-CoV-2 in a patient without symptoms of COVID-19 disease. ‘Mild’ disease was defined in the presence of a positive RT-qPCR test for SARS-CoV-2 in a patient with any COVID-19-related symptoms (e.g., fever, cough, sore throat, malaise, headache, body/muscle pain, nausea/vomiting, diarrhea, loss of taste or smell) in the absence of tachypnea, shortness of breath, or abnormal findings on chest X-rays. ‘Moderate’ disease was defined in the presence of a positive RT-qPCR test for SARS-CoV-2 in a patient with evidence of lower respiratory tract disease as shown at physical examination (tachypnea, shortness of breath) or abnormal findings on chest X-rays, with an oxygen saturation (SpO_2_) level of ≥94% measured by a pulse oximeter. Clinical improvement was defined as a reduction of 2 or more points in the 0–10 NRS of the severity of symptoms [16,17].

### 2.7. Efficacy Endpoints

The primary efficacy endpoint was the reduction in viral load (day 4 vs. baseline) in the active treatment group (bromhexine plus SOC) as compared with the control group (SOC alone). Secondary efficacy endpoints were the reduction in viral load (day 7 vs. baseline and day 14 vs. baseline) in the two study arms; the proportion of patients with a negative RT-qPCR test for SARS-CoV-2 (Ct value > 35 in at least two of three genes) in the two study arms; the time to achieve a negative viral load from baseline in the two study arms; and the comparison of the clinical efficacy in the two study arms, including reduction in the severity of each symptom (0–10 NRS score) at days 4, 7, 14, and 28 as compared with baseline; proportion of patients with clinical improvement and time to clinical improvement; proportion of patients with disappearance of each symptom at days 4, 7, 14, and 28, and time to disappearance; proportion of asymptomatic patients at days 4, 7, 14, and 28; proportion of patients requiring medical care, admission to the hospital, oxygen therapy, and development of complications related to COVID-19 disease over the study period; 28-day mortality rate; mortality rate after the end of study; and safety of bromhexine. 

### 2.8. Statistical Analysis

The null hypothesis was established as the absence of differences in the reduction of viral load after 4 days of starting treatment as compared with baseline (prior to treatment) between the two study groups. The sample size calculation for the primary efficacy endpoint was performed for a two-sided analysis of variance (ANOVA), with fixed effects and two levels in the factor evaluated corresponding to the active treatment or the control group. A type I error was set at a two-sided 0.05 level with a minimal effect with clinical relevance of 2 log10 reductions in viral copy number as the minimal difference between the on-treatment groups. A moderate effect of 0.25 (Cohen’s f) was targeted leading to an expected common standard deviation (SD) of 4 log10. Given a sample of 200 patients (100 assigned to bromhexine plus SOC and 100 assigned to SOC alone), a power of 94% was obtained to demonstrate the estimated difference (Sample Power, IBM-SPSS). The intention to treat (ITT) dataset (all randomized patients who received at least one dose of the study medication) was considered for efficacy and safety analysis.

The main analysis of the primary efficacy endpoint was measured by the Student’s *t*-test for independent samples. The ANOVA for repeated measurements and a factor (Split-Plot) with Bonferroni adjustment for multiple comparisons was applied to the comparison of viral load between the study groups at baseline, day 4, day 7, and day 14. The primary analysis was adjusted based on justified demographic and effect-modifying variables. Type I error was established at a two-sided 0.05 level. The software IBM-SPSS Version 27.0 (IBM Corp., Armonk, NY, USA) was used for the statistical analysis.

## 3. Results

### 3.1. Disposition of Patients

**Figure 1:**
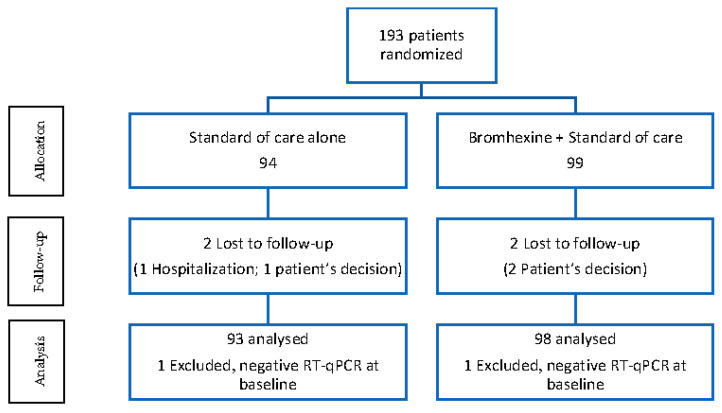
Flow chart of the study population. Analysis was based on the ITT dataset.

A total of 193 eligible patients were recruited by 19 participating centers and were randomized (99 to bromhexine plus SOC and 94 to SOC alone). However, one patient in each group was excluded because of a negative RT-qPCR test for SARS-CoV-2 at baseline. At follow-up, four patients (two in each study group) withdrew from the study, three of them because of the patients’ own decisions and one because of the need for in-patient care. The final evaluable ITT population included 191 patients, 98 in the bromhexine plus SOC group and 93 in the SOC alone group. The flow chart of the study population is shown in Figure 1.

### 3.2. Baseline Characteristics

A total of 127 patients were women (66.5%) and 64 men (33.5%), with a mean (SD) age of 47.8 (1.1) years. Almost all patients were Caucasian (93.7%) and 6.3% Hispanic. History of previous COVID-19 was recorded in 37 patients (19.4%), with a mean time elapsed from infection to enrollment in the study of 16.3 (1.4) months. A total of 182 patients (95.3%) had been vaccinated against SARS-CoV-2 and had received a mean number of doses of 2.4 (0.06), with a mean of 5.3 (0.2) months from the last vaccination dose. As shown in Table 1 differences in demographics, BMI, and data of previous SARS-CoV-2 infection between the study groups were not found.

In relation to the severity of COVID-19, 179 patients (95.9%) presented with mild disease, 7 (3.7%) with moderate disease, and 5 (2.6%) were asymptomatic. The distribution of patients according to the severity of disease was similar in the two study groups, with 1 (1%) and 4 (4.3%) asymptomatic patients, 94 (95.9%) and 85 (91.4%) patients with mild disease, and 3 (3.1%) and 4 (4.5%) patients with moderate disease in the bromhexine plus SOC and SOC alone groups, respectively.

The number of symptoms ranged between 0 and 19, with a mean of 6.1 (0.3) symptoms. The distribution of severity of symptoms was similar in the two study groups, although nasal congestion was significantly more severe in the SOC alone group than in the bromhexine plus SOC group (mean 6.4 [2.1] vs. 5.3 [2.0], *p* = 0.008); ear pain was also more severe in the SOC alone group (mean 7.0 [2.7] vs. 3.8 [1.6], *p* = 0.047). The results of the physical examination were similar in the two study groups (Table 2).

A total of 126 patients (66%) were receiving treatment for medical conditions at inclusion in the study. Statistical differences between the study groups were observed in the percentage of patients treated with bronchodilators (*p* = 0.033) and receiving symptomatic treatment (*p* = 0.034), which were higher in the SOC alone group, whereas treatment for concomitant diseases was higher in the bromhexine plus SOC group (*p* < 0.001). The administration of C (cardiovascular system) products and D (Dermatological) drugs was higher in the bromhexine plus SOC group (*p* = 0.002 and *p* = 0.012, respectively). The use of R (respiratory system) drugs was higher in the SOC alone group (*p* < 0.001).

The viral load was homogeneous between the study groups, with a mean (SD) Ct value of 22.5 (0.6) for ORF1ab, 22.8 (0.6) for N Protein, and 47.1 (2.4) for S Protein. The percentages of patients with low, medium, and high viral loads were 6.1%, 25.5%, and 68.4% for the bromhexine plus SOC group and 8.6%, 19.4%, and 72% for the SOC alone group.

### 3.3. Efficacy Endpoints

**Figure 2:**
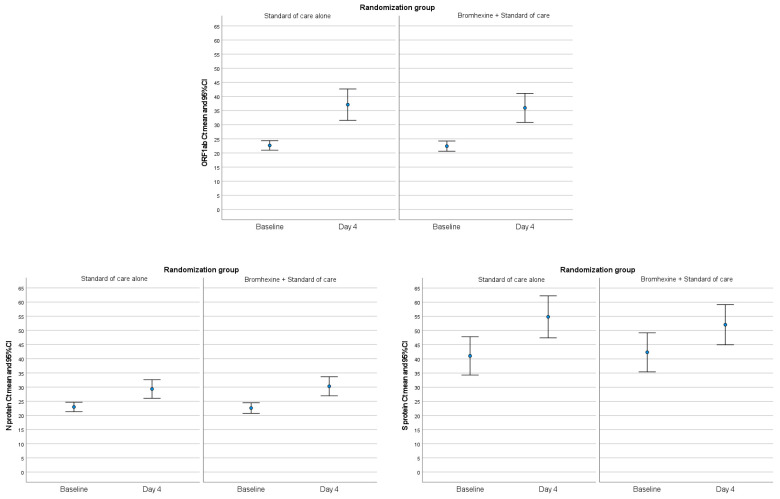
Reduction of viral load from baseline to day 4 of treatment for ORF1ab, N Protein, and S Protein in the two study groups. For all the comparisons Baseline versus Day 4, *p* value was <0.001.

Changes in viral load from baseline to day 4 were similar in the two study groups for the three specific SARS-CoV-2 genes (Figure 2). The mean Ct values for ORF1ab viral load were 13.54 (26.02) in the bromhexine plus SOC group as compared with 14.43 (26.94) in the SOC alone group (mean difference 0.89, 95% CI −6.67 to 8.45; *p* = 0.817). The mean Ct values of N Protein were 7.70 (18.47) in the bromhexine plus SOC group and 6.36 (17.05) in the SOC alone group, with a mean difference of -1.34 (95% CI −6.42 to 3.74; *p* = 0.603). For the S Protein, the mean Ct values were 9.74 (29.54) and 13.78 (26.81) for the bromhexine plus SOC and SOC alone groups, respectively, and a mean difference of 4.04 (95% CI −4.30 to 12.37; *p* = 0.340).

**Figure 3:**
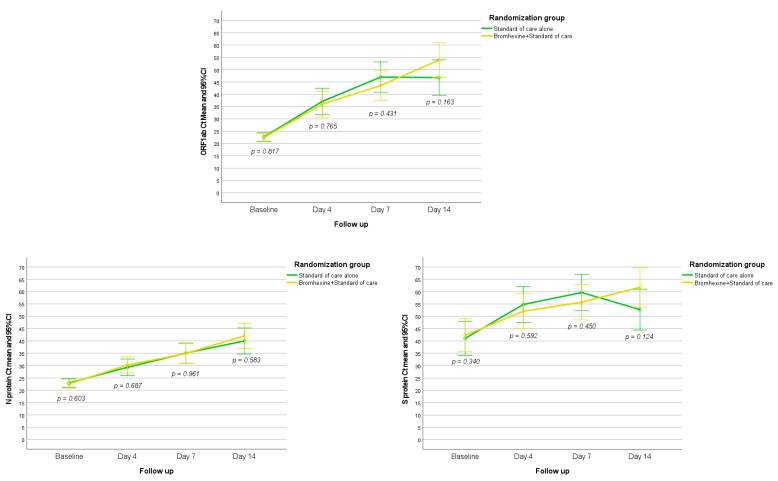
Evolution of Ct values of ORF1ab, N Protein, and S Protein at follow-up in the two study groups.

In the overall study population, Ct values of ORF1ab, N Protein, and S Protein increased significantly from baseline throughout days 4 to 14 (*p* < 0.001). For the comparison of Ct values of ORF1ab between the two study groups, there were no significant differences on day 4 (*p* = 0.765), day 7 (*p* = 0.431), and day 14 (*p* = 0.163). Similar findings were obtained for Ct values of N Protein at day 4 (*p* = 0.678), day 7 (*p* = 0.961), and day 14 (*p* = 0.583), as well as for Ct values of S Protein at day 4 (*p* = 0.592), day 7 (*p* = 0.450), and day 14 (*p* = 0.124) (Figure 3).

A sensitivity analysis performed on the primary efficacy endpoint and in the evolution of the viral load without imputation rules applied to the dataset showed no significant differences in the main efficacy results between the study groups.

No significant differences were found between bromhexine plus SOC and SOC alone in the percentage of patients with RT-qPCR positivity on day 4 (86.7% vs. 80.6%, *p* = 0.254), day 7 (74.5% vs. 65.6%, *p* = 0.179), and day 14 (53.1% vs. 61.3%, *p* = 0.251). Differences in the percentages of patients with low, medium, and high viral loads between the study groups at 4, 7, and 14 days were not found either. The median time to obtain an RT-qPCR negative result was 14 days (95% CI 12.2 to 15.8), without a significant difference between the study groups (*p* = 0.565).

No significant differences between the study groups were observed in the evolution of the vital signs that significantly improved from day 1 to day 28 (*p* < 0.05) in the oxygen saturation, heart rate (*p* < 0.01), and axillary temperature (*p* < 0.001). Also, there were no significant differences between the study groups in the severity of any of the symptoms observed throughout the study period, except for more intense dysgeusia in the SOC alone group than in the bromhexine plus SOC group (3 vs. 1.6 points, *p* = 0.005) and arthralgia (2.4 vs. 1.7 points, *p* = 0.014) on day 4. A total of 38 patients (19.9%) continued with persistent symptoms after day 28, with no differences between the study groups (Table 3).

**Figure 4:**
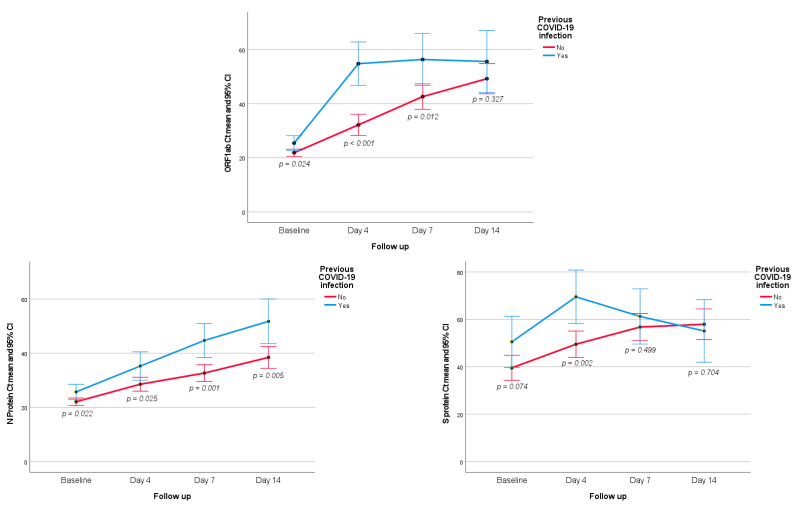
Evolution of ORF1ab, N Protein, and S Protein viral load according to the presence or absence of previous COVID-19 infection.

Patients with previous SARS-CoV-2 infection showed significant lower viral load at baseline and during the follow-up compared to patients with no previous COVID-19 (Figure 4). This difference was not observed on the vaccinated versus non vaccinated patients.

### 3.4. Safety Outcome

A total of 13 patients (6.8%) experienced adverse events, 8 patients in the bromhexine plus SOC group (8.2%) and 5 patients in the SOC alone group (5.4%), with no statistically significant difference (*p* = 0.445). The total number of adverse events observed was 17, 64.7% (*n* = 11) mild, and 23.5% (*n* = 4) moderate. A case of unrelated severe dizziness in one patient (5.9%) and another case of serious pulmonary thromboembolism (5.9%) were observed.

Three adverse events were considered related to bromhexine (dizziness, nausea, and pasty mouth), two possibly related (constipation and tinnitus), and one unknown (pruritus), with 11 adverse events unrelated to the study treatment (64.7%). No adverse event led to premature discontinuation of the study drug. Two moderate treatment-emergent laboratory abnormalities were observed in the bromhexine plus SOC group but were considered unrelated (leucocyte elevation, transaminase elevation).

At 12 days after the initiation of the study, one patient from the SOC alone group was required to be admitted to the hospital and oxygen therapy due to the worsening of COVID-19.

None of the patients died 28 days after completion of the study.

## 4. Discussion

This clinical trial explored the antiviral activity in clinical practice of an already marketed product, bromhexine, as drug repositioning in combination with standard of care. No differences were observed in the viral load at day 4 of the initiation of the study treatment in patients treated with bromhexine compared to those that only received standard of care.

Of the studies carried out in patients with COVID-19 registered in the Spanish Clinical Studies Registry (REEC) at the initiation date of the study, most of them were conducted in the hospital setting, in critically ill or moderately ill patients, and only 21.9% included patients mildly affected or asymptomatic patients. In fact, very few studies are being conducted in the outpatient setting, where there is a higher volume of patients with COVID-19. The higher proportion of studies in patients with moderate-to-severe disease is justified by the urgent need for treatments for patients at higher risk. However, the highest volume of patients infected with SARS-CoV-2 and capable of transmitting the disease are patients with mild symptoms and asymptomatic patients. These groups of patients are diagnosed, treated, and followed in primary care centers, the setting in which this study was conducted.

No previous clinical trials with bromhexine in patients with COVID-19 have been carried out in Spain, and there is limited evidence of the efficacy of this drug in the literature, the usefulness of which remains controversial [10,11,12,13,14]. Despite the recognition of the pharmaceutical properties of bromhexine to inhibit TMPRSS2 and its potential role in treating or preventing SARS-CoV-2 infection [10,16,18,19,20,21], expectations of efficacy in clinical practice appeared to be disappointing, which are consistent with findings of the present study.

In this randomized clinical study, the active treatment group (bromhexine plus SOC) was compared with a control group of SOC alone. A sample of 191 patients was included, 66.5% were women, with a mean age of 47.8 years, which is in agreement with overall data recorded in Spain with the most affected age range during the pandemic being between 50 and 59 years, with 55% of women [22]. The eligibility criteria established in the study limited the recruitment rate, since the groups of patients at higher risk of developing COVID-19 (e.g., older age, cardiovascular disease, COPD, cancer, immunosuppression, and other conditions) were excluded. The large majority of patients had mild disease, which accounted for a high mean number of clinical symptoms of 6.1 at initial presentation. Overall baseline data, including vital signs and distribution of symptoms, was similar in the two study groups, except for nasal congestion and ear pain, which were more severe in the SOC alone group. The prescription of bronchodilators and symptomatic treatments was more frequent in the SOC alone group, but differences for specific drugs were not found.

Viral load at baseline was similar in the two study groups as well as the percentages of patients with low, medium, and high viral loads. It was not possible to obtain the translation from Ct values to the number of viral copies, so comparison to viral loads reported in other studies is not possible. However, in other studies of bromhexine in hospitalized patients [12,13] or medical personnel [14], viral loads were not measured. In a protocol for systemic review and meta-analysis to assess the efficacy and safety of bromhexine hydrochloride tablets in treated pediatric COVID-19, assessment of viral load was not included among the types of outcome measures [23].

In relation to the primary efficacy endpoint of a reduction in the viral load from baseline to day 4, there were no differences between the study groups for none of the specific genes of the SARS-CoV-2 pathogenic viral RNA strain. In all three ORF1ab, N Protein, and S Protein genes, statistically significant reductions in viral loads were found from baseline to any time point of the follow-up for the overall study population, but differences at days 4, 7, and 14 between the study groups were not observed and these findings were confirmed in the sensitivity analysis. On the other hand, the percentage of patients with positive RT-qPCR results on days 4, 7, and 14 were similar in the two study groups, as was the percentage of patients classified into the groups of low, medium, and high viral loads. Patients with previous SARS-CoV-2 infection showed lower viral loads than patients without a history of COVID-19 disease, but differences between vaccinated and non-vaccinated patients were not found.

Other clinical data included the lack of differences between the study groups in the evolution of vital signs, overall improvement of severity of symptoms, and percentage of patients with persistent symptoms after day 28.

Regarding safety outcomes, a few patients reported adverse events without differences between the study groups. Most adverse events were of mild intensity and unrelated to treatment. In three cases (dizziness, nausea, and pasty mouth), adverse events were considered to be related to the use of bromhexine, and 2 cases (constipation and tinnitus) were possibly related. None of the patients discontinued the study because of any adverse event. One patient in the SOC alone group required in-patient care and oxygen therapy, with a successful recovery.

The open-label design is a limitation of the study. Although the primary efficacy endpoint was a laboratory variable on which a placebo effect is unlikely to occur, the inclusion of a control arm was important to determine whether there were differences in the evolution of the viral load when patients received the active medication, as well as to compare variables not considered in the study design that could influence the primary or secondary efficacy endpoints. In fact, we observed differences in the administration of concomitant drugs, with the use of bronchodilators and symptomatic treatments more frequently among patients in the SOC alone group. The relationship between these therapies and the reduction of viral load is unknown. In addition, the effect of bromhexine on the evolution of symptoms could not be evaluated due to the limited sample size. The SARS-CoV-2 virus variant responsible for the infection suffered by the study patients was not analyzed. The most frequent variant at the time of study completion in Spain was Omicron (100%) BA.5 and derivatives [24], but it has not been studied whether the mechanism of action of bromhexine might differ as per virus variant. So, it is unknown if the results could have been different if the study should be completed earlier in the pandemic.

## 5. Conclusions

In this study, treatment with bromhexine plus SCO was associated with a viral load reduction of ORF1ab, N Protein, and S Protein genes at day 4, which was not significantly different than similar viral load reductions observed with SOC alone. The present findings do not seem to provide arguments in favor of using bromhexine for treating patients with mild-to-moderate COVID-19 disease managed in the primary care setting although it can be used as a supplementary agent in addition to the standard treatment to reduce symptoms in these patients.

## Figures and Tables

**Table 1:** Demographic and previous SARS-CoV-2 infection in the study groups.

Variables	Total Patients (*n* = 191)		SOC Alone (*n* = 93)		Bromhexine + SOC (*n* = 98)		*p* Value
	N (%)	Mean (SD)	N (%)	Mean (SD)	N (%)	Mean (SD)	
Gender							
Male	64 (33.5)		34 (36.6)		30 (30.6)		0.384
Female	127 (66.5)		59 (63.4)		68 (69.4)		
Age, years		47.8 (1.1)		48.4 (1.5)		47.2 (1.6)	0.570
Race *							
Caucasian	179 (93.7)		88 (94.6)		91 (92.9)		0.615
Hispanic	12 (6.3)		5 (5.4)		7 (7.1)		
Body mass index, kg/m^2^		25.8 (0.4)		25.9 (0.6)		25.7 (0.5)	0.837
Previous COVID-19 infection							
No	154 (80.6)		75 (80.6)		79 (80.6)		0.995
Yes	37 (19.4)		18 (19.4)		19 (19.4)		
Severity of previous COVID-19 infection							
Asymptomatic *	1 (2.7)		1 (5.6)		0		0.511
Mild	22 (59.5)		12 (66.7)		10 (52.6)		
Moderate	11 (29.7)		4 (22.2)		7 (36.8)		
Severe	3 (8.1)		1 (5.6)		2 (10.5)		
Persistent	0		0		0		
Time from previous SARS-CoV-2 infection, months		16.3 (1.4)		16.0 (1.8)		16.6 (2.1)	0.831

* Fisher exact test was applied. SOC: standard of care; SD: standard deviation.

**Table 2:** Data of physical examination in the two study groups.

Variables	Total Patients (*n* = 191)		SOC Alone (*n* = 93)		Bromhexine + SOC (*n* = 98)		*p* Value
	N	Mean (SD)	N	Mean (SD)	N	Mean (SD)	
Systolic BP, mmHg	191	123 (1.0)	93	123 (1.0)	98	124 (1.0)	0.677
Diastolic BP, mmHg	191	76 (1.0)	93	76 (1.0)	98	76 (1.0)	0.740
Respiratory rate, breaths/min	191	16 (0)	93	16 (0)	98	16 (1.0)	0.473
Oxygen saturation, %	190	97 (0)	93	97 (0)	97	97(0)	0.624
Heart rate, beats/min	190	79 (1.0)	93	78 (1.0)	97	79 (1.0)	0.579
Axillary temperature, °C	190	36.5 (0.1)	93	36.5 (0.1)	97	36.4 (0.1)	0.703

SOC: standard of care; SD: standard deviation.

**Table 3:** Persistent symptoms observed after 28 days of follow-up in the two study groups.

Symptom	SOC Alone Group (*n* = 45) N (%)	Bromhexine + SOC Group (*n* = 52) N (%)	Total (*n* = 97) N (%)
Fever	0	1 (1.9)	1 (1.0)
Cough	9 (20.0)	5 (9.6)	14 (14.4)
Odynophagia	3 (6.7)	2 (3.8)	5 (5.2)
Dyspnea	3 (6.7)	1 (1.4)	4 (4.1)
Chest pain	0	1 (1.9)	1 (1.0)
Chills	1 (2.2)	1 (1.9)	2 (2.1)
Nausea	1 (2.2)	0	1 (1.0)
Vomiting	1 (2.2)	0	1 (1.0)
Diarrhea	2 (4.4)	2 (3.8)	4 (4.1)
Abdominal pain	0	2 (3.8)	2 (2.1)
Nasal congestion	6 (13.3)	7 (12.5)	13 (13.4)
Anosmia	2 (4.4)	4 (7.7)	6 (6.2)
Dysgeusia	2 (4.4)	1 (1.9)	3 (3.1)
Headache	3 (6.7)	4 (7.7)	7 (7.2)
Myalgia	1 (2.2)	3 (5.8)	4 (4.1)
Arthralgia	1 (2.2)	3 (5.8)	4 (4.1)
Weariness	6 (13.3)	6 (11.5)	12 (12.4)
Weakness	1 (2.2)	4 (7.7)	5 (5.2)
Anorexia	1 (2.2)	1 (1.9)	2 (2.1)
Dizziness	0	2 (3.8)	2 (2.1)
Depression	0	1 (1.9)	1 (1.0)
Conjunctival congestion	0	1 (1.9)	1 (1.0)
Pale, cold skin	1 (2.2)	0	1 (1.0)
Thrombotic phenomena	1 (2.2)	0	1 (1.0)

## Data Availability

Study data are available from the corresponding author upon reasonable request.
